# Experiences of living with symptomatic atrial fibrillation

**DOI:** 10.1002/nop2.1442

**Published:** 2022-10-30

**Authors:** Lena Holmlund, Karin Hellström Ängerud, Åsa Hörnsten, Fredrik Valham, Karin Olsson

**Affiliations:** ^1^ Department of Nursing Umeå University Umeå Sweden; ^2^ Department of Public Health and Clinical Medicine Umeå University Umeå Sweden

**Keywords:** atrial fibrillation, experiences, interviews, qualitative research

## Abstract

**Aim:**

To explore the experiences of living with symptomatic atrial fibrillation.

**Design:**

This study, with a descriptive qualitative design, was performed using semi‐structured individual interviews.

**Method:**

Six women and nine men with symptomatic atrial fibrillation were included. The transcribed interviews were analysed using qualitative content analysis. The COREQ checklist was followed.

**Results:**

The analysis resulted in a main theme, namely *balancing life* and included the themes *striving for illness control*, *becoming a receiver or an active partner in care* and *dealing with changed self‐image*. The participants strived to understand their illness, prevent attacks and manage anxiety. Some of the participants were not involved in decision‐making, were uninformed about self‐care measures, reported a lack of continuity in care and felt that the doctors focused on information about the medical part of care.

## 1 INTRODUCTION

Atrial fibrillation (AF) is the most common arrhythmia. In 2010, more than 33.5 million people worldwide were estimated to be affected and the disease continues to increase in both prevalence and incidence (Chugh et al., 2014). Increased prevalence is due to both improved awareness of AF resulting in systematic diagnosis of AF being made earlier (Kirchhof, Breithardt, et al., 2016) and increases in the life expectancy of the general population (Hindricks et al., 2020). Age is a major risk factor for AF, along with other factors that can be modified by lifestyle changes, namely high blood pressure, high cholesterol levels, diabetes, obesity and obstructive sleep apnoea syndrome (Hindricks et al., 2020).

## 2 BACKGROUND

The risk of AF for people of European descent and over the age of 55 is 1 in 3 (Hindricks et al., 2020). In Sweden, about 330,000 persons live with the diagnosis of AF (Riksförbundet HjärtLung, 2021). Since many individuals have asymptomatic AF and/or are undiagnosed, prevalence, in general, is assumed to be significantly higher (Kirchhof, Benussi, et al., 2016). Symptoms for those affected vary; some have no symptoms at all while others experience severe symptoms with a large symptom burden. Common symptoms are palpitations, shortness of breath and fatigue (Hindricks et al., 2020). Research shows that AF negatively affects health‐related quality of life (HRQoL), often in relation to the severity of symptoms (Freeman et al., 2015; Son et al., 2019). Increased severity of symptoms linked to AF may also be associated with increased depression and anxiety (Thompson et al., 2014). One review shows that patients with AF have lower HRQoL than both healthy persons and patients with other cardiovascular conditions (Son et al., 2019). A Swedish study demonstrates the existence of both physical and existential limitations in everyday life that negatively affect well‐being in patients with AF (Ekblad et al., 2013).

It is recommended that management of AF should include stroke prevention, symptom control and management of risk factors (Hindricks et al., 2020). It is also recommended that the patient should be involved, informed and empowered regarding the disease and self‐management (Kirchhof, Benussi, et al., 2016). However, previous research reports that patients lack advice and support on managing the disease (McCabe et al., 2011). Patients with newly diagnosed AF report that the information provided at consultations is difficult to understand and they additionally have difficulty being involved in decision‐making about their own care (Thrysoee et al., 2018). As previous research has shown, symptomatic AF affects several factors such as anxiety, depression, HRQoL and hospitalizations, research has also shown that it can be difficult to live with and manage the symptoms of AF. More research is needed to gain perspective from those who live with symptomatic AF in order to improve care. The purpose of this study was therefore to explore the experiences of living with symptomatic AF.

## 3 METHODS

### 3.1 Design and context

This interview study, with a qualitative design, was part of a larger main study that aimed to explore patients' experiences, quality of life and illness perceptions in AF. The main study recruited 180 participants with AF who were admitted for electrical conversion at a university hospital in Sweden.

### 3.2 Participants

In this study, 15 participants with symptomatic AF from the main study were included through purposive sampling to get variations in age, gender and experience of living with AF. Symptomatic participants were those who described any kind of symptoms related to AF at the time of inclusion in the study. Six women and nine men between 56 and 81 years of age (median 70), were approached by the second and last author by telephone and invited to the study, and all agreed to participate. They had lived with the disease for ½ to 10 years (median 3). They had varying levels of education, marital status and employment (Table 1). Additional inclusion criteria were a willingness to participate in interviews, being Swedish speaking and aged 18 years or older.

**TABLE 1:** Demographic and clinical characteristics of the study population

Adults with atrial fibrillation	15
Age in years, median (range)	70 (56–81)
Gender	
Women	6
Men	9
Years with AF, median (range)	3 (½–10)
Home situation	
Living alone	5
Married/partner	10
Education (highest level achieved)	
Elementary school	1
Senior high school	6
University	8
Employment	
Employed	3
Retired	12
Previous diagnoses of:	
Hypertension	8
Diabetes	1
Ischaemic heart disease	5
Heart failure	4
Preventive anticoagulation therapy	15
Antiarrhythmic drugs	15

*Note*: Except as indicated, data are presented as number of subjects.

### 3.3 Data collection

The individual interviews were semi‐structured and followed an interview guide developed by second, third and last author. The focus was on living with AF, specifically how the participants manage AF, their participation and decision‐making in care, their perception of the illness, and their future expectations. To get a deeper understanding, probing questions such as ‘Can you please tell me more?’ were asked. The interviews were conducted between June 2018 and April 2019 by the last and the second author and two nurses who were under specialist training. None of the interviewers was involved in the care of the participants. The interviews were held either in their home (*n* = 6) or at the hospital in a quiet and secluded room (*n* = 9) in accordance with each participant's wishes. The interviews lasted about 20 to 40 min (median 26) and were digitally recorded and later transcribed verbatim.

### 3.4 Data analysis

The analysis of the text followed qualitative content analysis with an inductive approach according to Graneheim and Lundman (2004). It is a method used to identify differences and similarities in experiences and to describe not only the manifest content of the text but also an interpretation of the latent content (Graneheim et al., 2017; Graneheim & Lundman, 2004; Lindgren et al., 2020). To get a sense of the whole, the transcribed text was read through several times. Text corresponding to the aim was identified. This text was divided into meaning units and condensed and coded upon content. Coding was completed manually. The codes were compared regarding similarities and differences and sorted into subthemes and themes on various levels of abstraction and interpretation. A main theme that captured the essence of the data was formulated. The interpretations in the various steps of the analysis were reflected on and discussed between the authors until consensus was achieved. Extensive experience in content analysis is represented in the research group.

### 3.5 Ethical considerations

This study followed the ethical principles outlined in the Declaration of Helsinki (‘World Medical Association Declaration of Helsinki: ethical principles for medical research involving human subjects’, 2013), and was approved by the Swedish Ethical Review Authority. The participants received both oral and written information about the study. Written informed consent was obtained from all participants. The potential benefits of this study were considered by the authors to outweigh the risks and burdens that participants might experience from participation.

### 3.6 Trustworthiness

Graneheim and Lundmans writing (2004) guided the striving to achieve trustworthiness. For credibility, there was a purposeful selection of participants to get variations in gender, age and living conditions and a range for the length of time people had lived with the disease. The focus was on the same questions in all the interviews to achieve dependability. For transferability, we have given clear descriptions of the data collection, characteristics of participants and context and the process of analysis. Along with the presentation of the results, representatives' quotes were used for demonstrating dependability (Graneheim et al., 2017).

## 4 RESULTS

The analysis yielded the main theme of *balancing life* and the following three themes derived from various subthemes: *striving for illness control*, *becoming a receiver or an active partner in care*, and *dealing with changed self‐image*. The main theme and the themes with subthemes are presented in Table 2 and in the sections that follow with quotations to promote a deeper understanding.

**TABLE 2:** Subthemes, themes and main theme

Subthemes	Themes	Main theme
Trying to understand the illness	*Striving for illness control*	*Balancing life*
Managing anxiety and symptoms		
Receiving information but seeking more	*Becoming a receiver or an active partner in care*	
Being more or less involved		
Transitioning between weak and strong	*Dealing with changed self‐image*	
Accepting changes and dependence on care		

### 4.1 Balancing life

The main theme *balancing life* illustrates the participants' various attempts to manage the entire life situation. It reflects how the participants tried to understand that they have AF and how they would manage the disease. It contains the participants' various attempts to navigate a fragmented care system and the lack of information, support and participation in the care that they experienced. It is about how the participants struggled to balance and keep life as normal as possible despite illness and a changed self‐image.

### 4.2 Striving for illness control

This theme concerns different ways of getting control over the illness with the desire of preserving ordinary life. It applies to both thoughts and action, to understand the illness and predict attacks as well as handling physical and mental manifestations.

#### 4.2.1 Trying to understand the illness

The participants had varying experiences of being affected by AF. They described a process that included an understanding that they were ill, understanding why they had AF and understanding what triggered the attacks.

For some, the first symptoms came suddenly, without warning. Others reported unclear symptoms, more of a feeling of discomfort and fatigue, making it difficult at first to understand what was happening to them. Participants stated that when searching for medical advice, not all doctors did a complete medical examination including control of heart rate and blood pressure, which could have delayed the diagnosis of AF.

Yes, the worst thing is that I did not understand that I had it. For a long time, I had difficulty walking and breathing and I was generally ill, but I never understood why. The healthcare centre did not seem to care much either. (p. 11)

Some formulated theories about the cause of their symptoms based on lifestyle and individual experiences. For example, there were theories of mental exhaustion, high blood pressure or stress. Individuals who were interested in exercise and trained intensively described their expectation that exercise would protect them from illness. When symptoms of AF appeared, they assumed that they had not trained enough and they responded by increasing it. Being fit may also have made it harder to understand that there was something wrong with their own body.

I got very high values on my bike heart rate monitor so I sent it to the producer. After 4 days I got a response saying ‘There is nothing wrong with your monitor, talk to your doctor!’ (p. 1)

Participants also wondered why they had developed this disease. Some had asked their doctor what could have caused their illness but they received no clear answer. They searched for explanations and found themselves lacking someone to talk to about their questioning. The demands of a job, inheritance, age and alcohol abuse were examples of suggested causes.

I may drink too much but no one in health care has talked about this. (p. 1)

Repeated attacks of AF created anxiety and irritation and most participants tried to understand what triggered the attacks. Some reported that the attacks were unpredictable; others identified connections to stress and anxiety, alcohol, hard work or infections.

#### 4.2.2 Managing anxiety and symptoms

The unpredictability of the attacks and the fact that AF concerns the heart often created anxiety, especially early in the disease. Treatment with antithrombotic drugs could also contribute to anxiety. In most instances, the participants did not share their anxieties with anyone else but tried to handle them on their own. Anxiety was sometimes more difficult to cope with than the physical symptoms, especially when it occurred at night or when living alone. Many expressed the belief that emotions such as anxiety and stress could aggravate their AF and since AF itself also caused anxiety, they tried to stay calm. Some managed by finding reasonable explanations for symptoms that did not frighten them or by calmly reasoning with themselves.

I'm talking to my heart: can't you take it easy, we're in bed, everything is calm all around, we do not have to be nervous about anything, nothing dangerous has happened. (p. 5)

They also tried to think positively, as in ‘I take antithrombotic and heart medication’ or ‘they will treat me’. Some bought heart rate monitors to get a sense of control; others instead managed anxiety by avoiding checking their pulse.

Several reported side effects of beta‐blockers such as fatigue, also describing difficulty in knowing whether tiredness was caused by the medicine or the disease. For those who had other illnesses as well, it was sometimes difficult to distinguish which illness was giving rise to the symptoms, resulting in uncertainty as to how to act.

I get stressed. That's the worst. It's connected to Parkinson's disease. But also atrial fibrillation, I think. Sometimes I do not know which is which because they affect each other … I'm not sure, I might even have atrial fibrillation right now. (p. 14)

Some used prescribed medication to interrupt attacks, which gave a sense of control: ‘I usually take a beta‐blocker, or two. I can eat them as candy’ (p. 1). Others made lifestyle changes to prevent or reduce attacks, such as, working less or reducing alcohol consumption. They reported that they had learned what their limit was, how much wine they could tolerate, or what activity to avoid. It was also important for them to manage the risks that the symptoms entailed. Those who had symptoms of dizziness were at risk of falling if they were careless. Participants stated that they had to think strategically, get to know their body's signals, and be prepared.

Physical symptoms often changed and became less severe. Many participants also described their anxiety decreasing over time. Trust in the healthcare system was of great importance for a reduction in symptoms.

Yes, in the beginning, I was quite worried and thought it was unpleasant and risky. But I have had three conversions and have at times been to hospital and it has passed by itself. The doctors have said that there is nothing really dangerous so I worry less and less about it now. (p. 10)

### 4.3 Becoming a receiver or an active partner in care

This theme concerns how the participants are informed about their disease and how they acquire more knowledge. It also concerns whether they become more or less involved in their own care and changes in that involvement over time. It also describes what difference it makes if people are involved or not.

#### 4.3.1 Receiving information but seeking more

The participants reported different experiences of receiving information. They generally stated that they had been well treated by skilled doctors who clearly informed them about which medications they recommended and the importance of taking them. Almost all had supplemented the given information by searching online to get a better understanding of the disease.

I have read online and tried to understand by myself and for me, it is quite important to feel that I understand what it is about and that I am informed as much as possible. It would have been valuable to have someone knowledgeable to call and ask questions, not because it would happen very often but still …. (p. 10)

Many expressed a desire to have someone to discuss their thoughts with. For example, they wanted to talk through issues related to lifestyle, exercise intensity, treatment options, the right time for ablation and the possibility that the heart may have been damaged by medications or treatments. Only a few reported that they had received lifestyle advice, with no one being told about the importance of weight loss.

Not really much advice and support. No one talks about what I should do to avoid this. I have not received anything like that, but they have said that I should live as usual. (p. 6)

Friends or relatives with medical knowledge were often consulted on opportunities for medical treatment or lifestyle advice.

#### 4.3.2 Being more or less involved

Living with AF is a process that leads to greater insight and impact over time. Participants shared that they had to be active themselves and ask questions to get more detailed information about aspects of the disease other than medication. One participant described how a doctor had tried to force on her medicine that she did not want. Others reported that they had had an impact on their care and had claimed their right to discontinue medications that produced side effects or to adjust the dosage. Some reported, though, that they trusted and followed the doctor's advice without questioning and were satisfied with that.

We sat and discussed—me, my wife and my doctor. I asked if I could remove that half tablet in the evening. “No”, he said, “not given the rise in the value”. And I feel confident in my doctor, who has done my check‐ups for several years. When he says I should continue with the medicine, then I continue. (p. 4)

As participants became more experienced some took the initiative in adjusting their medication, sometimes without any discussion with their doctor. Some reported that in dialogue with their doctor they could influence the timing of the next ablation. Some participants described themselves as very energized and proactive in participating in care and treatment planning.

I am a leader and used to getting information. I got the phone number of the expert on arrhythmia, a number I thought was good to have. We have had conversations where I have had the opportunity to discuss important issues. (p. 2)

The participants reported that the ability to change outcomes through improved lifestyles contributed to a sense of hope. Hope was also said to be a result of being involved in their care and having a plan for the future, including having a backup plan in case the first one did not work. For some, it was difficult to accept a pacemaker or an ablation, for example, but involvement in care planning gave them time to get used to the idea.

Participants with several diseases often experienced a lack of coordination between clinics with respect to changes in treatment or prior examinations that could involve the discontinuation of drugs. For this reason, they understood the importance of knowledge and involvement in their care.

The specialists have not been good at cooperating, but I do not know what to demand either. One doctor takes responsibility for one disease, and another for the other disease. And so they make a statement and … but no more than that. I have to make my own decisions. (p. 14)

### 4.4 Dealing with changed self‐image

This theme concerns the perception of self and perception by others. Because AF comes in attacks, physical ability varies over time, leading to difficulties in how to define yourself, as weak or strong, self‐managing or a person in need of support.

#### 4.4.1 Transitioning between weak and strong

Attacks of AF were reported to make them lose energy immediately: ‘I am an active person. Suddenly I lose all strength and I have difficulty breathing’ (p. 5). The difference electrical conversion made was expressed with this metaphor: ‘I felt like a newborn. Fresh air, blue skies and rippling water’ (p. 6). The abrupt changes seemed to be particularly difficult to accept for those who usually perceived themselves as strong. As one participant said: ‘I don't want to be a person without strength’ (p. 7). Nonetheless, change was a problem for all. Some had previously seen illness as a sign of weakness and the result of insufficient exercise. They in particular had difficulty understanding why they could be affected, expressing that they ‘did not deserve this’.

I have been wondering if the doctors really understand that I may not be a normal patient because practising sport is still very important to me after all. They could give counselling that is more individualized for me, preferably a doctor with their own experience of exercise. (p. 12)

Symptoms of AF could also have negative social consequences: ‘Social consequences of symptoms are almost the worst. I get sweaty and I don't feel fresh’ (p. 2).

Participants reported uncertainty in making plans. For those who had employment outside the house, the attacks could be inconvenient. Some avoided mentioning heart problems when they needed to be absent from work, to avoid scaring co‐workers. It was also stated that the symptoms gave feelings of inferiority, which could lead to avoidance of activities.

Last year I was free from attacks for a long time and was planning a trip. Then I caught a cold and the fibrillation returned. I was both angry and sad. (p. 2)

#### 4.4.2 Accepting changes and dependence on care

As time passed the participants had to accept changes in life, specifically that they could not manage as much as before and they had to live with uncertainty. Participants also realized that AF was a chronic disease with an ongoing need for medical care.

Once it has started, you are vulnerable all the time … I have learned that now I have to look at myself in a different way. You cannot do everything that you have done before. With some sadness, you realize that you have to give up certain things. But I think it is good in the end. In the end, you have to accept some changes. (p. 2)

Participants learned to cope with relapses and also to accept the need for repeated treatments to stop or prevent attacks. Most had undergone several cardioversions and some had undergone or been offered catheter ablation. Many reported that they had to accept increasing dependence on care but appreciated seeing the same professional team for repeated treatments, including nurses with extensive knowledge and effective management. This consistency provided security and helped in managing the disappointment of relapses. Participants who had met many different doctors stated that it would have been nice to have a doctor who had followed their entire course of illness. Those who had continuous contact with the same doctor were confident that they could have a discussion about, for example, medication adjustments and future treatment.

## 5 DISCUSSION

In the present study, the purpose was to explore the experience of living with symptomatic AF. The intention was to gain deeper insight into and understanding of what it means to live with the disease. The analysis resulted in the main theme, *balancing life*, which encompassed the themes of *striving for illness control, becoming a receiver or an active partner in care* and *dealing with changed self‐image*.

The participants described a variety of experiences of being affected by AF and different ways to manage symptoms, which in many cases were based on their own beliefs and experiences. Having symptoms of AF that could occur at any time caused problems with planning their lives, with the participants avoiding social activities because of the fear of having a new attack of AF. This led, in the long run, to negative social consequences, forgoing activities that they had previously enjoyed doing. This result is in line with previous studies (Altiok et al., 2015; Stridsman et al., 2019).

Some of the participants wanted healthcare staff to take responsibility for their care and felt secure in that decision. However, most participants wanted to get involved in their care and in planning for the future. Several concept analyses have been done regarding the concept of patient participation (Cahill, 1996; Castro et al., 2016; Sahlsten et al., 2008). In a previous study, it was concluded that patients describe patient participation in a broader sense than what healthcare staff and legislation do. Patients want knowledge, rather than being informed, and focus on interacting, rather than only taking part in the decision‐making (Eldh et al., 2010). Their results are more in line with the International Classification of Functioning, Disability and Health (ICF), where participation is defined as ‘involvement in a life situation’ (WHO, 2001, p. 10). This also corresponds to the results of our study, where participants expressed a desire for someone to exchange knowledge with and someone who understood their situation in addition to giving support. Participation in care presupposes a relationship, sharing information and knowledge as well as a mutual commitment to ongoing activities (Sahlsten et al., 2008). However, it is important to look at the patient's specific needs; there are differences in the extent to which patients want to be involved, as our results show. Furthermore, patients with AF with increased uncertainty about the disease and reduced levels of vitality and management in everyday life have lower confidence in decision‐making (Hedberg et al., 2018).

Due to fragmented care, with poor communication between the different caregivers, some participants in our study realized the importance of having a good knowledge of AF and the right information about their treatment, to ensure good quality of care. The lack of continuity and cohesive care was clearly illustrated in a previous Danish study, describing how the lack of communication between different caregivers made it difficult to get balanced information and support on when and where to seek care (Hoegh et al., 2015). The presence of several care providers can increase the risk of interruptions in the care chain, which can lead to the patient and caregivers seeing the patient's needs, resources and planning in different ways.

In this study, the participants described how their self‐image changed over time because of AF, from being a strong person to being weak and dependent on care. Altered self‐image in connection with the onset of chronic diseases has previously been described in the literature (Ambrosio et al., 2015; Charmaz, 1983). Ambrosio et al. describe learning to live with a chronic disease as going through a complex multidimensional process (Ambrosio et al., 2015). Healthcare professionals should be aware that the transition from feeling healthy to suddenly suffering from AF may affect a patient's self‐image. One study has shown that patients with a high level of understanding of their disease report greater acceptance of the disease, have fewer AF‐related symptoms, use more effective coping strategies, and have better control over the disease (McCabe, 2011). Accepting the disease affects HRQoL positively in patients (Jankowska‐Polańska et al., 2018). A negative perception of an illness can reduce the chances of recovery and may lead to an increased need for care (Petrie & Weinman, 2006). Informing patients newly diagnosed with AF that the disease is not immediately life‐threatening and does not require restrictions on everyday life could reduce fear and make patients more receptive to AF management (McCabe et al., 2020). Early intervention, soon after diagnosis, is important in helping the person through this transition with a focus on living as good a life as possible. Because the symptoms and experiences of AF typically differ from one person to another, the intervention should focus on the individual's experience, situation and knowledge in consultation with the patient.

The main theme of *balancing life*, shows that living with AF is not an easy task. Although there were differences in how AF was experienced by the participants, for everyone it meant a transition in life in which many factors affected how life would be going forward. Transitions often require individuals to change their definition of themselves in their social context, the result of new knowledge and adaptation to the new situation (Meleis, 2010). Some transitions can take place simultaneously, which increases the complexity (Meleis et al., 2000). In health care, it is part of the nurse's task to be aware of and help patients through transitions. Supporting patients through transitions is difficult. Given today's fragmented care, a change is therefore desirable, one in which care is based on a holistic view and based on the individual's situation and needs.

Given that the experience of living with AF differed between the participants, a uniform path in health care should not be proposed but rather one that adapts care to the unique person. A person‐centred approach to care can strengthen the patient's position and increase the patient's participation in their care. Studies have shown that person‐centred care can improve HRQoL (Brännström & Boman, 2014) as well as self‐efficacy, self‐care, dignity, mental and physical status, and can reduce health costs and symptom burden in patients with heart failure (Ulin et al., 2015). Implementation of person‐centred clinics for patients with AF can therefore improve the care for these patients.

## 6 METHODOLOGICAL DISCUSSION

A limitation of this study is that it was performed in a single hospital. The participants varied in age, the number of years living with the disease and living conditions. However, many participants had a high level of education, which may have affected the results. The research group has broad experience of patients with AF, which could have affected the interpretation of the participants' experience. Given the possibility of preconceived ideas arising from a prior understanding of AF, discussions about the interpretations of the results were held as a group. The fact that there were several interviewers and that interviews were held both at the hospital and in their homes, may have affected the results. By following an interview guide the risk of different content was minimized and there were no major differences in content or length of interviews regardless of where the interviews were conducted.

## 7 CONCLUSION

Balancing life entails persons with AF having to handle both symptoms and anxiety. Transitioning between strength and weakness and accepting being dependent on care might lead to a changed self‐image. To be co‐actors in their own care, patients need to be engaged in issues of importance for them, and in creating both short‐ and long‐term goals with healthcare professionals. Accomplishing these outcomes requires continuity and sensitivity from healthcare professionals. There is a need to continue to develop care for patients with AF and to let a person‐centred approach permeate all aspects of care.

## 8 RELEVANCE TO CLINICAL PRACTICE

The experience of living with AF differs between individuals, but for everyone, AF leads to a change, from not having the disease to living with the disease, which in most cases is chronic. To facilitate the transition, healthcare efforts need to be tailored to meet the needs of each unique person, especially soon after diagnosis. A person‐centred form of care may enable greater patient involvement, thereby aiding the transition.

## AUTHOR CONTRIBUTIONS

The study was designed by Karin Hellström Ängerud and Karin Olsson and they also contributed to data collection. The analysis was conducted by Lena Holmlund, Åsa Hörnsten and Karin Olsson. All authors contributed to analysis and interpretation of the results. Lena Holmlund and Karin Olsson drafted the first version of the manuscript, which was revised critically for important intellectual content by all authors. The final version of the manuscript was approved for submission by all authors.

## FUNDING INFORMATION

This work was supported by the Heart Foundation of Northern Sweden, Strategic Research Area Health Care Science (SFO‐V), Region of Västerbotten and Umeå University.

## CONFLICT OF INTEREST

The authors declared no conflicts of interest.

## Data Availability

The data that support the findings of this study are available from the corresponding author upon reasonable request.
